# Fibrinous Coagula in the Heart

**Published:** 1864-04

**Authors:** 


### FIBRINOUS COAGULA IN THE HEART.

The two following articles have appeared editorially in the *Chicago Medical Journal*, and are suggestive of so much, both upon the subjects of Heart Clot, and the use of veratrum viride, that we take our first opportunity to make the re-print:

"FIBRINOUS COAGULA IN THE HEART.—There is a great deal of scattered experience with regard to this interesting subject which should be collected. Dr. J. F. MINER, Editor of the *Buffalo Medical and Surgical Journal*, in an interesting article entitled, "Intra-Cardiac Blood Concretion," adduces two cases going to show the presence of these formations in death from chloroform. He makes these pertinent suggestions:

"It is perhaps an entirely new view of the dangers of chloroform to regard it as liable to produce death by inducing blood-clot during the periods of suspended respiration which so often occur under its influence; still, in connection with hæmorrhage, which is supposed also to favor this formation, it may have more influence than generally supposed. Whatever interrupts the circulation, or greatly enfeebles it, must certainly be regarded as more or less operative in producing such result."

In which connection we beg leave to call Dr. Miner's attention to our own remarks, under this head, on page 332 (July No.) of this volume.—Any notable reduction of the heart's action directly favors formation of these coagula, and their subsequent danger. The therapeutic and prophylactic indications are obvious. Not only death, but "complications" in the way of local disorders, are exceedingly liable to occur from the formation and detachment of these coagula into the current of the circulation. We repeat: "He who boasts that with veratrum viride, or tartar emetic, or other sedative, he has reduced the pulsation from a hundred, or more, below the normal standard, by no means convinces me that, unwittingly perhaps, he is not responsible for the sudden, or it may be slow and lingering, death which ensues." The attempt to control pulsation should, mainly, if not wholly, be through endeavor to remove the cause, whatever that may be. The heart labors more rapidly in consequence of the demand made upon it, just as its muscularity increases when there is permanent obstruction. The hypertrophy is not disease—the more rapid stroke is not disease! This thing is worth looking after.

The following is the article above referred to:

Fibrinous Coagula in the Heart.—The fibrinous coagula occasionally found in the cavities of the heart, and which have long been considered by the greater number of authorities as merely formed *in articulo mortis*, have latterly—since the scientific investigations with reference to Thrombi and Emboli, have thrown so much light on a previously obscure branch of pathology—attracted much attention from pathologists.

At a recent meeting of the London Pathological Society, Dr. Ogle exhibited eight preparations "illustrating the spontaneous formation of fibrinous coagula, at a long period before death, in the cavities of the heart, most of which had undergone considerable softening, and some of which were quite fluid in their centre. In several of these specimens the central puriform fluid was bounded by a firm, smoothish surface, reminding one of the wall of an abscess, and welled out on section of the clot being made. The firm character of these coagula, their color, their adherence to the walls of the heart, and the changes which had taken place or were taking place within them, marked conclusively that the formation of the coagula had occurred sometime, possibly some weeks, before death. Out of the eight cases, this old standing and degenerating coagulum was found in the right auricle in five cases, in the right ventricle in three cases, and in the left ventricle in one case only. In almost all instances the cases had been such as included retardation of the blood's circulation through the lungs, and mostly a long and lingering death; the patients also being chiefly subjects of ill-health or intemperance."

Dr. Ogle thought pyæmia might be produced by bursting of a concretion. No special phenomena had marked the formation; a term of months might have elapsed he believed in some cases. Dr. Ogle also related some cases of embolism of both middle cerebral arteries, and of the coronary arteries.

We have frequently observed these fibrinous concretions in the cavities of the heart, and in at least one instance seen distinct vascularity of the new formation entitling it to the denomination of polypus.

Our friend Dr. J. P. Linn, exhibited to us, a few days since, a well marked specimen, evidently formed long anterior to the death of a well-known resident of this city.

With regard to the causes, it is evident that all that is requisite is first a nidus upon which the clot may form, then an excess of the fibrin of the blood; and lastly, but perhaps most important of all, such a degree of retardation of the velocity of the circulation as may favor deposit upon the roughened surface of the excess of fibrin present.

The physiological principles involved are well understood, and have been taken advantage of, again and again, in the cure of aneurism and stoppage of hæmorrhage by position, and mechanical and medical appliances.

Propositions to which we wish now to call attention are simply and briefly these: *The increased frequency of the heart's contraction in inflammatory and febrile affections has the physiological object of preventing the formation of coagula in its cavities. Rash interference with this prophylactic effort of nature, by agents which notably retard the heart's action, directly favors formation of these coagula and their subsequent dangers.*

He who boasts that with veratrum viride, or tartar emetic, or other sedative, he has reduced the pulsation from a hundred or more, below the normal standard, by no means convinces me that, unwittingly perhaps, he is not responsible for the sudden, or it may be slow and lingering death which ensues. In this respect, it must be confessed, tartar emetic is immensely superior to its proposed substitutes, because it contemporaneously with its action upon the heart, defibrinates the blood.

